# Efficacy of Telmisartan Plus Amlodipine in Nonresponders to CCB Monotherapy

**DOI:** 10.1155/2013/627938

**Published:** 2013-06-17

**Authors:** Steen Neldam, Dingliang Zhu, Helmut Schumacher

**Affiliations:** ^1^Rodøvre Centrum 294, 2610 Rodøvre, Denmark; ^2^Shanghai Ruijin Hospital, Shanghai Institute of Hypertension, 197 Ruijin 2nd Road, Shanghai, China; ^3^Boehringer Ingelheim Pharma GmbH & Co. KG, Binger Strasse 173, 55216 Ingelheim am Rhein, Germany

## Abstract

Hypertensive patients unable to reach blood pressure (BP) targets with antihypertensive monotherapy may be switched to a combination of two medications with complementary modes of action for improved treatment response. This *post hoc* analysis pools data from 2812 patients, 1891 of whom were not at goal (diastolic BP [DBP] <90 mm Hg) with amlodipine 5 mg (A5) monotherapy who subsequently switched to telmisartan 40 or 80 mg (T80)/A5 single-pill combination (SPC) or amlodipine 10 mg (A10) monotherapy, and considers an additional 921 patients, 616 of whom were not at goal with A10 monotherapy who switched to telmisartan/amlodipine SPC. Patients switched to telmisartan/amlodipine SPC achieved significantly greater BP reductions compared with continued monotherapy (*P* < 0.0001) with reductions of −15.2/−10.9 mm Hg seen with T80/A5 after 8 weeks in patients switched from A5. BP goal (<140/90 mm Hg), systolic BP goal (<140 mm Hg), and DBP goal (<90 mm Hg) were reached by significantly more patients with telmisartan/amlodipine than with monotherapy (*P* < 0.0001 for all comparisons; 56.1%, 69.7%, and 66.9%, resp., in patients who switched from A5 to T80/A5). Early use of such combination therapy should be considered to quickly reach BP targets, particularly in patients with added risk.

## 1. Introduction

Hypertension is a major risk factor for cardiovascular (CV) and cerebrovascular morbidity and mortality [1]. Achieving blood pressure (BP) goal rapidly, as well as sustaining this level of control, is important to reduce CV risk and prevent organ damage [2]. Treatment guidelines generally recommend treatment of hypertension with a BP goal <140/90 mm Hg for the majority of patients. More aggressive targets of <130/80 mm Hg in patients with diabetes or renal disease have also been suggested [3, 4], although such low BP targets have been recently questioned following inconclusive results from several trials [2, 5–7]. Despite these recommendations, less than one-third of patients taking hypertensive treatment reach the desired goal of <140/90 mm Hg [8], and many of these will be taking only one antihypertensive agent. 

Combining two drugs from different classes with complementary mechanisms of action is likely to result in additional reductions in BP compared with either agent used alone [9, 10]. For this reason and based upon evidence from many antihypertensive studies, most guidelines agree that the majority of patients need combination therapy to achieve BP goals, and so initial combination therapy is now more frequently recommended [3, 4, 11, 12]. Single-pill combinations (SPCs) can also help overcome the poor treatment adherence that has been associated with free combination therapies with multiple antihypertensive agents, administered at different time intervals, and, therefore, patients are more likely to reach and maintain their BP goal and reduce their CV risk [13]. A calcium channel blocker (CCB) combined with a renin-angiotensin system (RAS) inhibitor (such as an angiotensin II receptor blocker [ARB] or an angiotensin-converting enzyme inhibitor) is one such complimentary combination. Indeed, based on outcomes from the avoiding cardiovascular events through combination therapy in patients living with systolic hypertension (ACCOMPLISH) trial, in which a RAS inhibitor/CCB was superior to a RAS inhibitor/diuretic [14], this combination was particularly effective in reducing CV risk and is recommended by guidelines [3, 4, 12]. 

Telmisartan is the longest acting of all ARBs with a half-life of 24 hours [15] and has been shown to reduce CV risk [16], leading to an indication for the reduction of CV morbidity in patients with manifest atherothrombotic CV disease (history or coronary heart disease, stroke, or peripheral arterial disease) or type 2 diabetes mellitus with documented target organ damage [15]. When combined with the CCB, amlodipine, additive BP lowering is evident, compared with the individual monotherapies [17, 18]. This telmisartan/amlodipine combination has also been demonstrated to be effective in patients at all stages of hypertension, as well as in those with added risk factors including obesity, diabetes, or metabolic syndrome [19–24].

Several published studies have demonstrated the efficacy of the telmisartan/amlodipine combination in patients uncontrolled on amlodipine monotherapy [20, 25]; however, a pooled analysis incorporating a large population is currently lacking. This paper presents a *post hoc* analysis of data from the Boehringer Ingelheim clinical trials database comparing telmisartan/amlodipine in combination therapy versus amlodipine monotherapy in patients who were uncontrolled with amlodipine alone. Where appropriate, data was pooled across studies and analyzed.

Only trials from the Boehringer Ingelheim database were chosen as no other relevant studies could be found using other databases. This also enabled access to patient-level data and to ensure consistency in the recording of outcomes.

## 2. Methods

### 2.1. Studies

The Boehringer Ingelheim trial database was searched for all studies investigating the telmisartan/amlodipine SPC therapy in hypertensive patients uncontrolled on any CCB monotherapy. Four randomized, double-blind studies, completed between September 2008 and August 2011, were identified, and these are detailed in Table 1. All studies included a 6–8-week amlodipine monotherapy (5 or 10 mg) run-in period, and patients who had not reached the diastolic BP (DBP) goal of ≥90 mm Hg following this run-in period were then randomized to amlodipine monotherapy or telmisartan/amlodipine SPC therapy for a further 8 weeks. Three of the studies (telmisartan/amlodipine single-pill study to assess the efficacy in patients with essential hypertension not controlled on A5 [TEAMSTA-5], T40/A5 in A5 nonresponders, and T80/A5 in A5 nonresponders) included patients uncontrolled on amlodipine 5 mg (A5) at the end of the run-in period of 6 weeks, and the fourth study (telmisartan/amlodipine single-pill study to assess the efficacy in patients with essential hypertension not controlled on A10 [TEAMSTA-10]) included patients uncontrolled on amlodipine 10 mg (A10) at the end of the run-in period. In the TEAMSTA-10 study, the run-in period was longer as patients began treatment with A5 for 2 weeks before being uptitrated to A10 for a further 6 weeks. 

During the randomized phases of the studies, the treatment regimens used were A5, A10, telmisartan 40 mg (T40)/A5, telmisartan 80 mg (T80)/A5, T40/A10, and T80/A10. In all studies, patients took their trial treatments once daily, in the morning. Seated BP was measured using a standard, validated, and calibrated sphygmomanometer. BP was measured at baseline (week 0), week 4, and week 8 in the three studies involving patients uncontrolled on A5, at baseline, and at weeks 2, 6, and 8 in the one study that included patients uncontrolled on A10. The change from reference baseline (i.e., after amlodipine monotherapy) in seated trough DBP was the primary endpoint in all four studies.

### 2.2. Statistical Analysis

Due to the similarities in trial design, data from the three trials in patients uncontrolled on A5 were pooled and analyzed. However, data from the one study in patients uncontrolled on A10 were analyzed separately.

The mean changes in seated DBP and systolic BP (SBP) from reference baseline, adjusted for baseline BP and study (where appropriate), were calculated, and treatments were compared using analysis of covariance. Other measures included BP goal (<140/90 mm Hg), DBP goal (<90 mm Hg), and SBP goal (<140 mm Hg) attainment rates, in addition to response rates for DBP (<90 mm Hg or a reduction ≥10 mm Hg) and SBP (<140 mm Hg or a reduction ≥20 mm Hg). The goal and response rates were compared between treatments using logistic regression adjusted for baseline BP and study. 

## 3. Results

### 3.1. Demographics

A total of 2812 patients were included in this analysis—1891 not achieving DBP goal <90 mm Hg on A5 monotherapy and 921 not achieving the same DBP goal on A10. For the analysis in patients not at goal on A5 monotherapy, groups were generally well matched for most demographic characteristics (Table 2). However, the T80/A5 group had slightly more females (39.9% compared with 34.7–36.7% in the other groups); the A5 and T40/A5 groups had higher proportions of Asian patients (69.9% and 60.0%, resp.) than the A10 and T80/A5 groups (20.3% and 49.1%), due to the inclusion of two Asian studies that did not contain A10 as a treatment. The baseline SBP in the A10 group was lower than in the other groups (140.9 mm Hg versus 147.2–147.9 mm Hg). The baseline SBP in the A10 group was lower than in the other groups (140.9 mm Hg versus 147.2–147.9 mm Hg). Considering those patients not at goal on A10 monotherapy, the groups were very well matched, with only the A10 group having fewer females (41.3%) compared with the T40/A10 and T80/A10 groups (47.1% and 46.1%, resp.) (Table 2).

### 3.2. Change in DBP/SBP from Baseline

**Figure 1:**
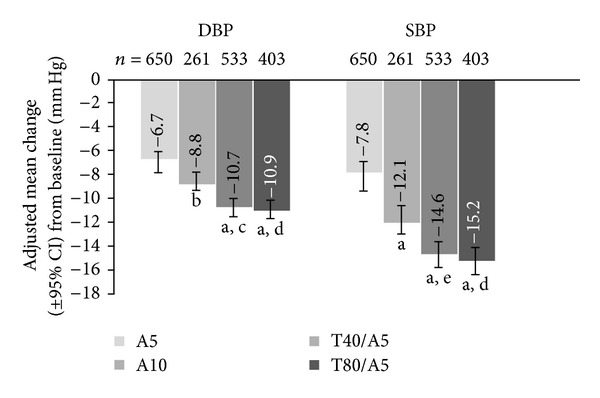
Adjusted mean change in DBP and SBP at week 8 from baseline in patients uncontrolled on A5 monotherapy. A5: amlodipine 5 mg; A10: amlodipine 10 mg; CI: confidence interval; DBP: diastolic blood pressure; SBP: systolic blood pressure; T40: telmisartan 40 mg; T80: telmisartan 80 mg. ^a^ *P* < 0.0001 versus A5; ^b^ *P* = 0.0003 versus A5; ^c^ *P* = 0.0011 versus A10; ^d^ *P* = 0.0005 versus A10; ^e^ *P* = 0.0032 versus A10.

All patients achieved significantly greater reductions in DBP and SBP when switched to telmisartan/amlodipine SPC therapy, compared with patients who continued on monotherapy (*P* < 0.0001). After 8 weeks of randomized treatment, patients not at DBP goal (<90 mm Hg) on A5 monotherapy who were maintained on A5 achieved DBP changes (from reference baseline) of −6.7 mm Hg (95% confidence interval [CI], −7.2, −6.1), and those who were uptitrated to A10 achieved DBP reductions of −8.8 mm Hg (95% CI, −9.8, −7.8; *P* = 0.0003 versus A5). Those patients who switched to T40/A5 and T80/A5 achieved DBP reductions of −10.7 mm Hg (95% CI, −11.4, −10.0; *P* < 0.0001 versus A5; *P* = 0.0011 versus A10) and −10.9 mm Hg (95% CI, −11.6, −10.1; *P* < 0.0001 versus A5; *P* = 0.0005 versus A10), respectively. Adjusted mean changes in SBP from baseline at 8 weeks were −7.8 mm Hg (95% CI, −8.6, −6.9) for patients maintained on A5 monotherapy, −12.1 mm Hg (95% CI, −13.6, −10.6; *P* < 0.0001 versus A5) for patients uptitrated to A10 monotherapy, −14.6 mm Hg (95% CI, −15.7, −13.6; *P* < 0.0001 versus A5; *P* = 0.0032 versus A10) for patients switched to T40/A5, and −15.2 mm Hg (95% CI, −16.3, −14.0; *P* < 0.0001 versus A5; *P* = 0.0005 versus A10) for patients switched to T80/A5 (Figure 1).

**Figure 2:**
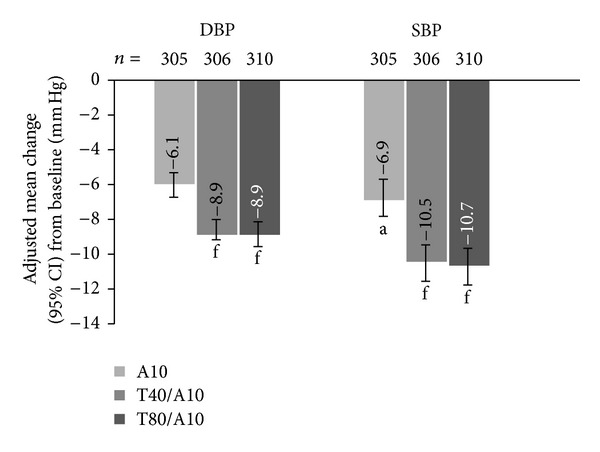
Adjusted mean change in DBP and SBP at week 8 from baseline in patients uncontrolled on A10 monotherapy. A10: amlodipine 10 mg; CI: confidence interval; DBP: diastolic blood pressure; SBP: systolic blood pressure; T40: telmisartan 40 mg; T80: telmisartan 80 mg. ^a^ *P* < 0.0001 versus A5; ^f^ *P* < 0.0001 versus A10.

In those patients who had not reached goal (DBP < 90 mm Hg) with A10 monotherapy, DBP was reduced by −6.1 mm Hg (95% CI, −6.8, −5.4) when maintained on A10 for a further 8 weeks; −8.9 mm Hg when switched to T40/A10 (95% CI, −9.6, −8.1; *P* < 0.0001 versus A10); and −8.9 mmHg when switched to T80/10 (95% CI, −9.6, −8.2; *P* < 0.0001 versus A10). Adjusted mean changes in SBP from baseline at 8 weeks were −6.9 mm Hg (95% CI, −7.9, −5.8) for patients remaining on A10 monotherapy, −10.5 mm Hg (95% CI, −11.6, −9.5; *P* < 0.0001 versus A10) for patients switched to T40/A10, and −10.7 mm Hg (95% CI, −11.8, −9.7; *P* < 0.0001 versus A10) for patients switched to T80/A10 (Figure 2).

### 3.3. Goal Attainment Rates

**Figure 3:**
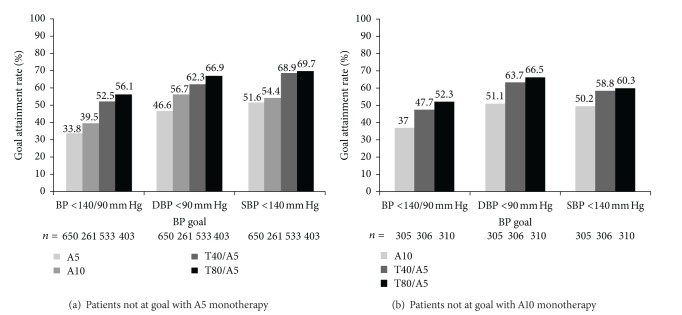
BP goal attainment rates at week 8. A5: amlodipine 5 mg; A10: amlodipine 10 mg; BP: blood pressure; DBP: diastolic blood pressure; SBP: systolic blood pressure; T40: telmisartan 40 mg; T80: telmisartan 80 mg.

A greater proportion of patients not at DBP goal (<90 mm Hg) with amlodipine monotherapy achieved the goals of BP < 140/90 mm Hg, DBP < 90 mm Hg, and SBP < 140 mm Hg when switched to telmisartan/amlodipine SPC therapy for 8 weeks compared with those who continued with amlodipine monotherapy (Figures 3(a) and 3(b)). Indeed, patients who switched to the telmisartan/amlodipine SPC therapy were significantly more likely to achieve goals than patients maintained on monotherapy (Table 3). 

In those patients who had not previously achieved goal with A5 monotherapy, DBP goal (<90 mm Hg) was achieved by only 46.6% of patients remaining on A5 and 56.6% of patients uptitrated to A10 compared with 62.3% patients who were switched to T40/A5 and 66.9% of patients who were switched to T80/A5. Similarly, SBP goal (<140 mm Hg) was achieved by 51.6% of those patients remaining on A5 monotherapy and 54.4% of those uptitrated to A10 compared with 68.9% of patients switched to T40/A5 and 69.7% who were switched to T80/A5. Overall BP goal (<140/90 mm Hg) was achieved by only 33.8% of patients remaining on A5 monotherapy and 39.5% of patients uptitrated to A10 compared with 52.5% and 56.1% of patients who switched to T40/A5 and T80/A5, respectively (*P* < 0.0001 for overall treatment differences of all comparisons).

In patients not at goal with A10 monotherapy, DBP < 90 mm Hg was achieved in only 51.1% of patients remaining on A10 compared with 63.7% of patients switched to T40/A10 and 66.5% of patients switched to T80/A10 (*P* = 0.0002 for overall treatment differences). SBP < 140 mm Hg was achieved in 50.2% of those remaining on A10 monotherapy compared with 58.8% switched to T40/A10 and 60.3% switched to T80/A10 (*P* = 0.0012 for overall treatment differences). Overall BP goal (<140/90 mm Hg) was achieved by only 37.0% of patients remaining on A10 monotherapy compared with 47.7% and 52.3% of patients who switched to T40/A10 and T80/A10, respectively (*P* < 0.0001 for overall treatment differences).

### 3.4. Response Rates

Patients not at goal with amlodipine monotherapy who received telmisartan/amlodipine SPC therapy were significantly more likely to achieve response targets than patients maintained on either monotherapy (Table 4). 

Response rates (DBP < 90 mm Hg or a reduction ≥10 mm Hg or SBP < 140 mm Hg or a reduction ≥20 mm Hg) were higher in patients randomized to telmisartan/amlodipine SPC therapy compared with response rates of patients who continued amlodipine monotherapy. In patients not at goal on A5 monotherapy, DBP response was achieved in only 51.6% of patients remaining on A5 and 62.5% of those uptitrated to A10 compared with 70.0% patients on T40/A5 and 73.0% of patients on T80/A5 (*P* < 0.0001 for overall treatment differences). SBP response was achieved in 57.8% of those remaining on A5 and 63.6% of those uptitrated to A10 compared with 74.3% on T40/A5 and 77.7% on T80/A5 (*P* < 0.0001 for overall treatment differences). In patients not at goal on A10 monotherapy, DBP response was achieved in only 53.4% of patients remaining on A10 compared with 66.0% patients on T40/A10 and 68.7% of patients on T80/A10 (*P* = 0.0002 for overall treatment differences). SBP response was achieved in 54.1% of those remaining on A10 compared with 64.7% on T40/A10 and 65.8% on T80/A10 (*P* = 0.0013 for overall treatment differences).

### 3.5. Adverse Events

In patients not at goal with A5 monotherapy, those who continued on A5 monotherapy experience a similar rate of drug-related adverse events and discontinuations due to adverse events compared with those who switched to either SPC dose (Table 5). However, those who switched to A10 monotherapy experienced numerically more drug-related adverse events and discontinuations due to adverse events. In patients not at goal with A10 monotherapy, the rates of respective AEs were similar in all three treatment groups. The number of serious adverse events reported in all groups was very low, irrespective of run-in medication. Interestingly, peripheral oedema mainly occurred in the European studies (A5 and A10 nonresponder trials), whereas in the Asian studies (T40/A5 in the A5 nonresponder trial and T80/A5 in the A5 nonresponder trial), peripheral oedema was not reported as frequently. In the A5 nonresponder trial, peripheral oedema was reported as follows: A10: 74 (26.8%), A5: 22 (8.2%), T40/A5: 14 (5.1%), and T80/A5: 10 (3.6%). In the A10 nonresponder trial, the respective numbers were A10: 22 (7.0%), T40/A10: 21 (6.7%), and T80/A10: 27 (8.5%).

## 4. Discussion

In this analysis of 2812 patients who failed to reach DBP goal (<90 mm Hg) with amlodipine monotherapy after 6–8 weeks of treatment, use of telmisartan/amlodipine SPC therapy was associated with significantly greater reductions in DBP and SBP compared with maintenance on, and uptitration of, amlodipine monotherapy. This is to be expected due to the complimentary modes of actions of the two drugs. Adding an ARB to CCB therapy should promote arterial and venous dilation by blocking the RAS system and attenuate renal hyperfiltration and peripheral edema induced by CCBs. In addition, the negative sodium balance promoted by CCBs may further reinforce the antihypertensive effect of the ARB [28–30].

The greatest treatment differences noted, 7.4/4.2 mm Hg, were between continuation on A5 monotherapy and switching to T80/A5. Slightly smaller treatment differences of 2.8–3.8 mm Hg were observed when patients not at goal with A10 switched to SPC therapy compared with those who continued with A10 monotherapy. This may be expected as patients who are unresponsive to higher doses of monotherapy are often considered more difficult to treat. These small differences in BP may be clinically relevant, as a large meta-analysis found that reductions in SBP even as small as 2 mm Hg reduced mortality from stroke by 10% and mortality from other CV causes by 7% in middle-aged individuals [31].

In this analysis, patients switched to SPC therapy were more likely to respond to treatment and achieve BP goal than those maintained on monotherapy. In patients not at goal with A5 monotherapy, the odds ratio (OR) of achieving BP goal (<140/90 mm Hg) was 3.43 (95% CI, 2.54, 4.64) for T80/A5 versus continuing A5 therapy, and for DBP goal (<90 mm Hg), the corresponding OR was 3.00 (95% CI, 2.22, 4.07). However, approximately half of all patients not at goal (DBP < 90 mm Hg) on either dose of amlodipine monotherapy after 6–8 weeks of treatment achieved DBP goal following a further 8 weeks of continued monotherapy treatment, and 56.7% of patients achieved DBP goal on switching to more potent monotherapy (uptitration to A10). This suggests that some patients initially unresponsive to monotherapy may eventually achieve BP goal if continued on the same treatment at the same or a higher dose of monotherapy. However, the time taken to achieve BP goal is also an important risk factor [32–36]. Therefore, due to greater likelihood of goal attainment and greater SBP/DBP reduction, early treatment with SPC therapy may be preferable to quickly achieve and maintain BP goal.

The BP reductions observed with telmisartan/amlodipine SPC therapy in patients not at goal with amlodipine monotherapy are similar to those observed with other ARB/CCB combinations. For example, a study investigating olmesartan/amlodipine in patients with moderate-to-severe hypertension, not at goal with amlodipine monotherapy, adds further support for switching to combination therapy [37]. Following 8 weeks of A5 monotherapy, nonresponders were randomized to receive either placebo plus A5 or a combination of olmesartan (10, 20, or 40 mg) plus A5 for a further 8 weeks. Adjusted mean changes in DBP versus placebo/amlodipine therapy were −2.0 mm Hg (*P* = 0.0207) for olmesartan 10 mg/A5, −3.7 mm Hg (*P* < 0.0001) for olmesartan 20 mg/A5, and −3.8 mm Hg (*P* < 0.0001) for olmesartan 40 mg/A5. Adjusted mean changes in SBP were −3.5 mm Hg (*P* = 0.0103), −5.8 mm Hg (*P* < 0.0001), and −7.1 mm Hg (*P* < 0.0001), respectively [37]. 

This is the first pooled analysis of telmisartan/amlodipine SPC therapy in a large number of patients (*n* = 1891) not at goal with A5 monotherapy, in addition to the retrospective analysis of another study (*n* = 921) in patients not at goal with A10 monotherapy. Limitations of these analyses are that they are retrospective and incorporate Boehringer Ingelheim-sponsored studies only. However, the endpoints stipulated in the individual trials are mostly identical to those used in this pooled and retrospective analysis, and results from the two separate analyses are similar. In addition, the use of the Boehringer Ingelheim studies enabled the study of patient-level data. In each study, criteria for randomization was DBP > 90 mm Hg. A significant proportion of randomized patients (15.8–30.2%) already had SBP < 140 mm Hg, which partly confounds the analysis of SBP data; however, the analysis of DBP is robust. The results are clinically relevant in terms of the doses investigated and the study design, investigating BP goal attainment with combination therapy in patients not at goal with monotherapy. 

## 5. Conclusion

Patients not at goal with A5 or A10 monotherapy achieved significantly greater DBP and SBP reductions compared with continuing with amlodipine monotherapy, and the majority of patients achieved BP goal when switched to telmisartan/amlodipine SPC therapy. Reported adverse events with SPC therapy were similar to or less than those experienced with continued monotherapy. Therefore, the early use of combination therapy, such as the telmisartan/amlodipine SPC, may be considered as an approach to quickly reach BP targets. This is of particular relevance to the more difficult-to-treat, added-risk, hypertensive patients.

## Figures and Tables

**Table 1:** Details of trials identified for inclusion into the analysis.

Trial	Description	Patient numbers	Study duration (weeks)	Run-in treatment (weeks)	Randomized treatments (8 weeks)
NCT00558428 Neldam et al. 2011 [20]	A5 nonresponder trial (TEAMSTA-5)	1057	8	A5 (6)	A5, A10, T40/A5, T80/A5
NCT00558064 Boehringer Ingelheim data on file [26]	T40/A5 in A5 nonresponder trial	520	8	A5 (6)	A5, T40/A5
NCT01103960 Boehringer Ingelheim data on file [27]	T80/A5 in A5 nonresponder trial	314	8	A5 (6)	A5, T80/A5
NCT00553267 Neldam et al. 2011 [25]	A10 nonresponder trial (TEAMSTA-10)	921	8	A5 (2), A10 (6)	A10, T40/A10, T80/A10

A5: amlodipine 5 mg; A10: amlodipine 10 mg; T40: telmisartan 40 mg; T80: telmisartan 80 mg; TEAMSTA-5: telmisartan/amlodipine single-pill study to assess the efficacy in patients with essential hypertension not controlled on A5; TEAMSTA-10: telmisartan/amlodipine single-pill study to assess the efficacy in patients with essential hypertension not controlled on A10.

**Table 2:** Demographic and baseline data.

	A5 run-in				A10 run-in		
	A10	A5	T40/A5	T80/A5	A10	T40/A10	T80/A10
Number of patients	261	671	533	426	305	306	310
Age, years mean (SD)	54.3 (10.7)	54.4 (10.2)	55.4 (10.4)	53.8 (9.8)	56.4 (10.4)	57.7 (9.4)	55.4 (9.8)
BMI, kg/m^2^ mean, (SD)	28.6 (4.9)	27.1 (4.8)	27.5 (5.0)	28.4 (5.0)	30.2 (4.4)	29.6 (4.4)	30.7 (4.9)
Female, %	35.2	36.7	34.7	39.9	41.3	47.1	46.1
Race							
Asian, %	20.3	69.9	60.0	49.1	0	0	0.6
Black, %	2.3	0.6	0.6	0.9	0	0	0.3
White, %	76.6	29.5	39.2	49.8	100	99.3	98.7
Other, %	0.8	0	0.2	0.2	0	0.3	0
Missing, %	0	0	0	0	0	0.3	0.3
Diabetes %	8.0	12.4	15.0	8.7	12.1	11.4	14.8
Baseline DBP, mean (SD)	96.5 (4.8)	96.4 (5.5)	96.4 (5.1)	96.7 (5.1)	95.6 (4.0)	95.5 (4.0)	95.6 (4.1)
Baseline SBP, mean (SD)	149.0 (11.8)	147.5 (12.0)	147.2 (12.3)	147.9 (11.9)	146.8 (10.2)	148.1 (9.4)	147.4 (9.4)
Duration of hypertension, years, mean (SD)	6.5 (8.0)	6.9 (7.8)	6.0 (7.4)	6.7 (7.8)	8.1 (7.1)	8.1 (7.7)	8.0 (7.5)

A5: amlodipine 5 mg; A10: amlodipine 10 mg; BMI: body mass index; DBP: diastolic blood pressure; SBP: systolic blood pressure; SD: standard deviation; T40: telmisartan 40 mg; T80: telmisartan 80 mg.

**Table 3:** OR for goal attainment and response rates in patients not at goal with A5 monotherapy.

Goal or response target (mm Hg)	A10 versus A5 OR (95% CI)	T40/A5 versus A5 OR (95% CI)	T80/A5 versus A5 OR (95% CI)
BP < 140/90	2.13 (1.49, 3.05)	3.07 (2.34, 4.04)	3.43 (2.54, 4.64)
DBP < 90	2.24 (1.58, 3.19)	2.62 (2.00, 3.44)	3.00 (2.22, 4.07)
DBP < 90 or reduction ≥ 10	2.12 (1.53, 2.96)	2.71 (2.10, 3.51)	2.73 (2.05, 3.65)
SBP < 140	1.94 (1.35, 2.81)	2.99 (2.24, 4.00)	3.25 (2.37, 4.48)
SBP < 140 or reduction ≥ 20	1.90 (1.36, 2.68)	2.69 (2.06, 3.52)	2.86 (2.11, 3.90)

A5: amlodipine 5 mg; A10: amlodipine 10 mg; BP: blood pressure; CI: confidence interval; DBP: diastolic blood pressure; OR: odds ratio; SBP: systolic blood pressure; T40: telmisartan 40 mg; T80: telmisartan 80 mg.

**Table 4:** OR for goal attainment and response rates in patients not at goal with A10 monotherapy.

Goal or response target (mm Hg)	T40/A10 versus A10, OR (95% CI)	T80/A10 versus A10, OR (95% CI)
BP < 140/90	1.78 (1.27, 2.52)	2.08 (1.48, 2.94)
DBP < 90	1.70 (1.22, 2.37)	1.95 (1.40, 2.73)
DBP < 90 or reduction ≥ 10	1.69 (1.22, 2.36)	1.93 (1.39, 2.70)
SBP < 140	1.78 (1.25, 2.54)	1.77 (1.25, 2.52)
SBP < 140 or reduction ≥ 20	1.70 (1.22, 2.38)	1.73 (1.24, 2.42)

A10: amlodipine 10 mg; BP: blood pressure; CI: confidence interval; DBP: diastolic blood pressure; OR: odds ratio; SBP: systolic blood pressure; T40: telmisartan 40 mg; T80: telmisartan 80 mg.

**Table 5:** Adverse event data.

	A5 run-in				A10 run-in		
	A10	A5	T40/A5	T80/A5	A10	T40/A10	T80/A10
Number of patients treated*	276	693	546	437	315	315	317
Patients with investigator defined drug-related AEs (%)	77 (27.9)	43 (6.2)	32 (5.9)	27 (6.2)	27 (8.6)	25 (7.9)	31 (9.8)
Patients with AEs leading to discontinuation (%)	21 (7.6)	9 (1.3)	6 (1.1)	5 (1.1)	7 (2.2)	9 (2.9)	4 (1.3)
Patients with SAEs (%)	1 (0.4)	4 (0.6)	3 (0.5)	1 (0.2)	1 (0.3)	3 (1.0)	0 (0.0)

*Note that AE data are reported for all patients treated. This is slightly higher than the number of patients with BP measurements available and reported in the efficacy analysis.
